# Effects of acute low-moderate dose ionizing radiation to human brain organoids

**DOI:** 10.1371/journal.pone.0282958

**Published:** 2023-05-31

**Authors:** Foluwasomi A. Oyefeso, Gabriela Goldberg, Nana Yaa P. S. Opoku, Marcelo Vazquez, Antonella Bertucci, Zhong Chen, Charles Wang, Alysson R. Muotri, Michael J. Pecaut

**Affiliations:** 1 Department of Biomedical Engineering Sciences, School of Medicine, Loma Linda University, Loma Linda, California, United States of America; 2 Department of Pediatrics, School of Medicine, University of California San Diego, La Jolla, California, United States of America; 3 Departments of Pediatrics and Cellular & Molecular Medicine, School of Medicine, Center for Academic Research and Training in Anthropogeny (CARTA), Kavli Institute for Brain and Mind, Archealization Center (ArchC), University of California San Diego, La Jolla, California, United States of America; 4 Center for Genomics, School of Medicine, Loma Linda University, Loma Linda, California, United States of America; 5 Department of Basic Sciences, School of Medicine, Loma Linda University, Loma Linda, California, United States of America; 6 Department of Radiation Medicine, School of Medicine, Loma Linda University, Loma Linda, California, United States of America; National Center for Toxicological Research, UNITED STATES

## Abstract

Human exposure to low-to-moderate dose ionizing radiation (LMD-IR) is increasing via environmental, medical, occupational sources. Acute exposure to LMD-IR can cause subclinical damage to cells, resulting in altered gene expression and cellular function within the human brain. It has been difficult to identify diagnostic and predictive biomarkers of exposure using traditional research models due to factors including lack of 3D structure in monolayer cell cultures, limited ability of animal models to accurately predict human responses, and technical limitations of studying functional human brain tissue. To address this gap, we generated brain/cerebral organoids from human induced pluripotent stem cells to study the radiosensitivity of human brain cells, including neurons, astrocytes, and oligodendrocytes. While organoids have become popular models for studying brain physiology and pathology, there is little evidence to confirm that exposing brain organoids to LMD-IR will recapitulate previous *in vitro* and *in vivo* observations. We hypothesized that exposing brain organoids to proton radiation would (1) cause a time- and dose-dependent increase in DNA damage, (2) induce cell type-specific differences in radiosensitivity, and (3) increase expression of oxidative stress and DNA damage response genes. Organoids were exposed to 0.5 or 2 Gy of 250 MeV protons and samples were collected at 30 minute, 24 hour, and 48 hour timepoints. Using immunofluorescence and RNA sequencing, we found time- and dose-dependent increases in DNA damage in irradiated organoids; no changes in cell populations for neurons, oligodendrocytes, and astrocytes by 24 hours; decreased expression of genes related to oligodendrocyte lineage, astrocyte lineage, mitochondrial function, and cell cycle progression by 48 hours; increased expression of genes related to neuron lineage, oxidative stress, and DNA damage checkpoint regulation by 48 hours. Our findings demonstrate the possibility of using organoids to characterize cell-specific radiosensitivity and early radiation-induced gene expression changes within the human brain, providing new avenues for further study of the mechanisms underlying acute neural cell responses to IR exposure at low-to-moderate doses.

## Introduction

The risks of radiation exposure can be divided into three categories: acute (occurring within the first few days after exposure), early delayed (occurring within a few weeks or months after exposure), and late delayed (occurring within several months or years after exposure) [1, 2]. Acute radiation syndrome (ARS) describes the pathological changes that occur within days or weeks after exposure to ionizing radiation (IR) [3]. The biological effects of IR depend on many factors, including dose, dose-rate, energy, exposure time, radiation quality and the radiosensitivity of different cell types [4, 5]. Unlike non-ionizing radiation, IR carries enough energy to remove electrons from atoms and creates free radicals—unstable atoms or molecules with highly reactive unpaired electrons—in a process known as an ionization event [6]. Thus, IR exposure presents a risk to human health when biomolecules (i.e. DNA, lipids, and proteins) become ionized and lead to chain reactions of ionization events in the body [7]. DNA damage and deficiencies in DNA repair can contribute to diseases by altering cell function, accelerating cell senescence, and promoting carcinogenesis [8]. Moreover, the production and accumulation of free radicals in tissue can exceed the detoxification of these products by protective antioxidants, giving rise to a pathological state known as oxidative stress [9]. Persistent oxidative stress is known to be associated with a range of chronic neurological diseases, including cancer, neurodevelopmental deficits, and dementia [9].

Experimental, clinical, and epidemiological studies provide strong evidence that exposure to moderate-high dose IR promotes neurobiological alterations caused by mitochondrial dysfunction and genomic instability, inhibiting differentiation of neural precursor cells, triggering apoptosis, and activating pro-inflammatory mechanisms [10–13]. Furthermore, studies have shown different vulnerabilities to IR and oxidative stress in cell- and region-specific manner [14, 15]. For example, vulnerability to atrophy varies in different regions of the cerebral cortex when exposed to high protracted doses of IR delivered in several 1.8–2 Gy fractions [15]. Although controversial, there are some reports that seem to indicate that a single exposure could be neuroprotective or therapeutic [16–18]. Others have identified cognitive and neurodevelopmental deficits in humans exposed to doses as low as 1 Gy in adults and 0.1 Gy in children [19–21]. The cellular effects of low-moderate doses of radiation are often subclinical but may significantly affect brain function and structure over time [19–21]. The accumulation of IR-induced DNA damage *in utero* can lead to mutations in neural progenitor cells that manifest as childhood conditions such as xeroderma pigmentosum and Cockayne Syndrome [22]. Despite the fact that terminally differentiated (mature) neural cells are less radiosensitive than neural stem/precursor cells and the adult brain is generally considered less radiosensitive than other organs, recent studies indicate that cell cycle arrest, synaptic remodeling, demyelination, chronic inflammation, cell migration, and altered gene expression induced by low-moderate dose IR exposure at the brain cellular level can be detrimental to overall health and worsen quality of life throughout adulthood [5, 23–25]. We chose protons as the source of IR because proton therapies are widely used for treating tumors of the central nervous system, and their neurobiological effects on healthy brain structures have not yet been fully characterized. Additionally, astronauts will be exposed to chronic and acute proton radiation doses during low-Earth orbit and deep space flight activities, so it is imperative to understand the effects of protons on neural cells.

Although traditional studies with human brains have yielded a great deal of useful information, there are still significant limitations to study radiation-induced damage using human brain tissue, thus limiting the development of treatment approaches and radiation countermeasures [26, 27]. In addition, many factors are difficult to control when using human brain tissue, such as standardizing methods for picking the correct premortem agonal period, elapsed time from death to tissue fixation, and time of fixation when analyzing postmortem brain tissue [19]. While animal models have contributed greatly to our understanding of the human brain, these protocols can be time-consuming, expensive, and experimental results can be misinterpreted due to stress caused by animal handling [28, 29]. Furthermore, genetic, phenotypic, anatomical, and physiological differences between human and model animals limit our ability to translate results from animal studies to humans [12, 30, 31]. Several influencing factors which contribute to controversial or contradictory findings from animal studies have been comprehensively discussed [32]. Traditional monolayer cell culture techniques also have a long history in research, and they allow researchers to study individual cell behavior and changes in gene expression. Cells can be cultured directly on flasks or an extracellular matrix coating with uniform access to nutrients and secrete signaling factors [28]. However, these cultures are limited in functional complexity and lack biologically relevant three-dimensional (3D) cytoarchitecture and signaling gradients which play important roles *in vivo* [33]. To overcome these limitations, brain organoids have become increasingly popular as a model for developmental, disease, and treatment studies [34, 35]. Through directed differentiation, these three-dimensional cell aggregates can develop the cellular diversity and early architectural features of specific brain regions during neurodevelopment, which increase in complexity as organoids develop in cultures [36–40]. The field of organoid research has seen significant advancements since the early development of these models, such as the presence of a blood-brain-barrier, myelinating oligodendrocytes, vasculature, microglia, multi-subunit structures with functional circuits, optic structures, and complex electrophysiological network dynamics [41–48]. As research advances, organoids continue to complement previous findings, bridge the gap between bench studies and clinical trials, and reduce animal use for research.

In this study, we used three-dimensional (3-D) brain organoids induced from human induced pluripotent stem cells (hiPSCs) to study the acute radiation responses of human brain cells in 3-D. We generated brain organoids from hiPSCs using a previously published method to promote the differentiation and maturation of neurons, astrocytes, oligodendrocytes, and other neural subtypes found within the cerebral cortex [46]. Here, we use histological staining and transcriptomic analysis of brain organoids to characterize the effects of a 0.5 or 2 Gy dose of 250 MeV protons at 30 minutes, 24 hours, and 48 hours after irradiation.

## Results

### Generation of brain organoids and characterization of irradiated brain organoids

**Fig 1:**
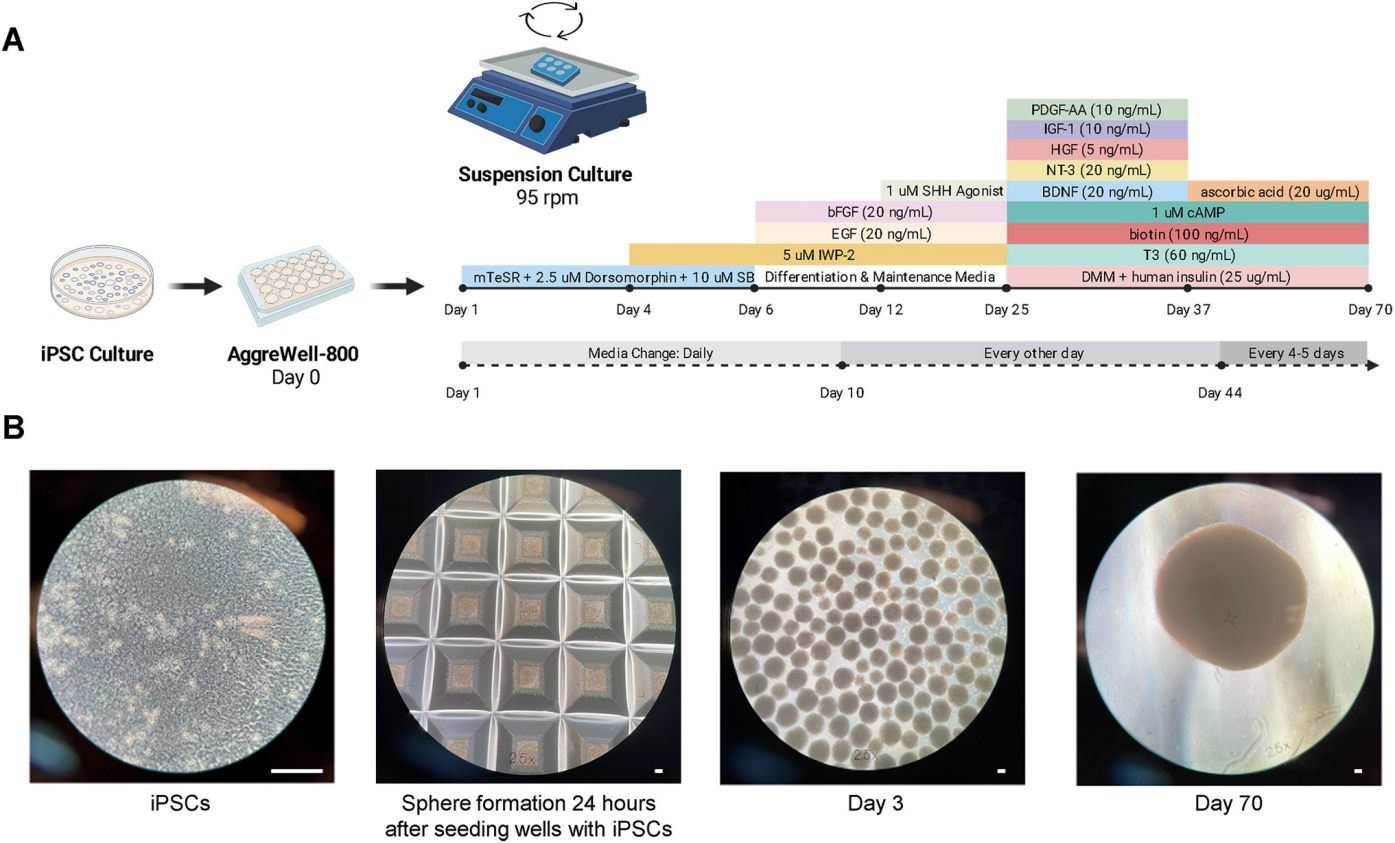
Generation and characterization of brain organoids. (A) Diagram illustrating the process of generating brain organoids from hiPSCs for up to Day 70 of suspension culture. The illustration was created with BioRender.com. (B) The process of generating brain organoids is shown at four stages, from hiPSC monolayer culture to spheroid formation, suspension culture of Day 3 organoids, and suspension culture of Day 70 organoids. The scale bar is 100 μm.

After 70 days in culture, the average diameter of the organoids was 2.4 mm (Fig 1). Prior to irradiation, all organoids expressed SOX2 and NESTIN (neural stem cells); NEUN, NFH, beta-tubulin III, and MAP2 (neurons); GFAP (astrocytes); OLIG2 and MBP (oligodendrocytes) (S1 Fig).

**Fig 2:**
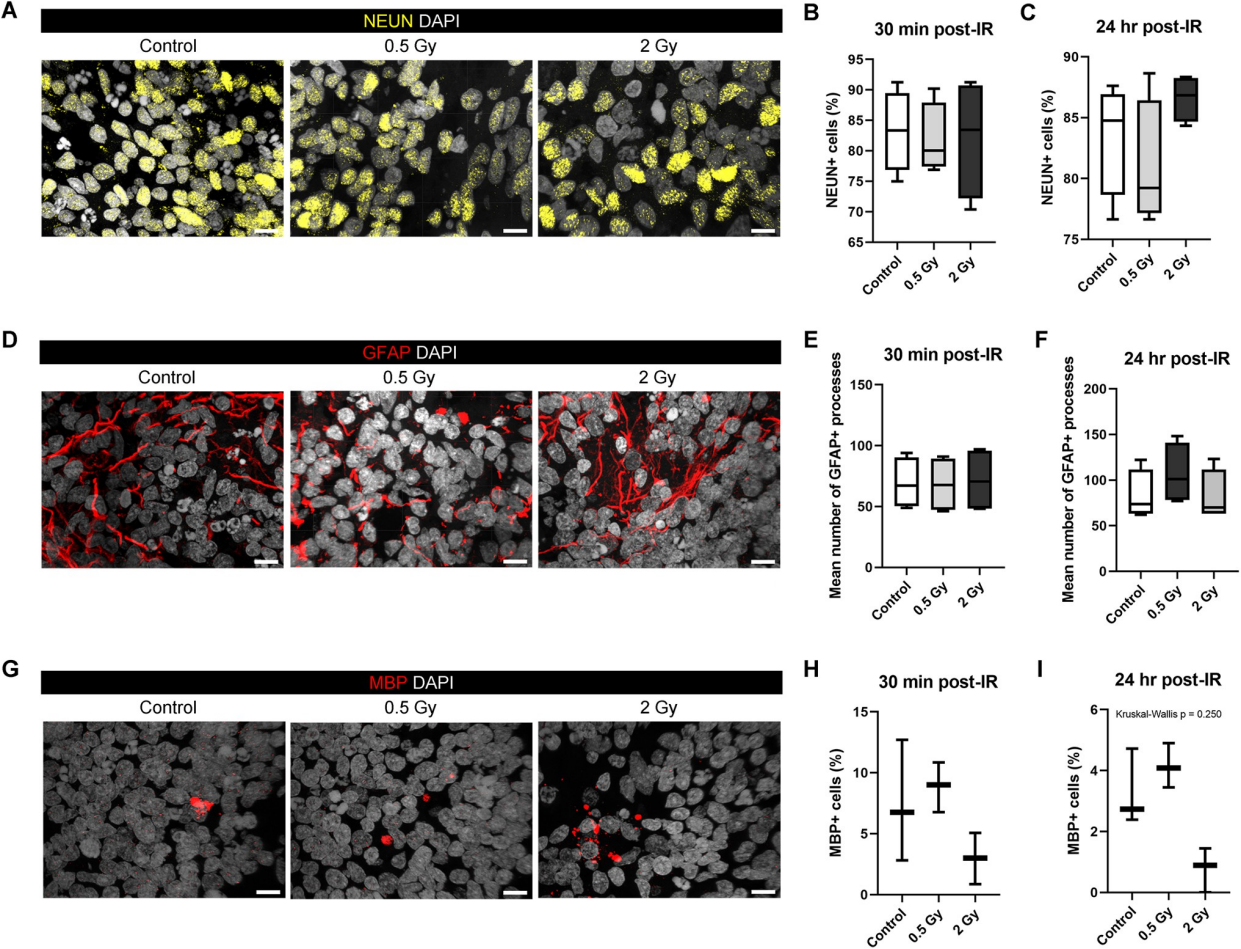
Radiosensitivity of different brain cell types after irradiation with a 0.5 Gy or 2 Gy dose of 250 MeV protons. Immunostaining of (A) NEUN+ neurons, (D) GFAP+ astrocytes, and (G) MBP+ oligodendrocytes in Day 70 WT83 C6 organoids 24 hours after irradiation with 0.5 Gy and 2 Gy doses as well as a control group. Kruskal-Wallis test (P < 0.05) with Dunn’s multiple comparison test was conducted for control versus 0.5 Gy versus 2 Gy collected 30 minutes (n = 4 per group for B and E; n = 3 per group for H) or 24 hours (n = 4 per group for C and F, n = 3 per group for I) after irradiation. Box-and-whisker plots show the range and median of cell counts across regions of interest. Scale bars = 10 μm.

Quantitative analysis of immunofluorescence (IF) stained WT83 C6 organoid sections showed no significant difference in the number of NEUN+ cells between the unirradiated control group and irradiated organoids at either 30 minutes or 24 hours after exposure (Fig 2A). Additionally, no significant differences were found in the number of GFAP+ astrocytic processes (Fig 2D) or MBP+ cells (Fig 2G) between unirradiated and irradiated organoids at either time point, though we did identify a strong trend indicating differences in MBP+ cell counts between 0.5 and 2 Gy irradiated organoids.

### Assessment of DNA damage in brain organoids 30 minutes after exposure to 0.5 or 2 Gy of 250 MeV protons

**Fig 3:**
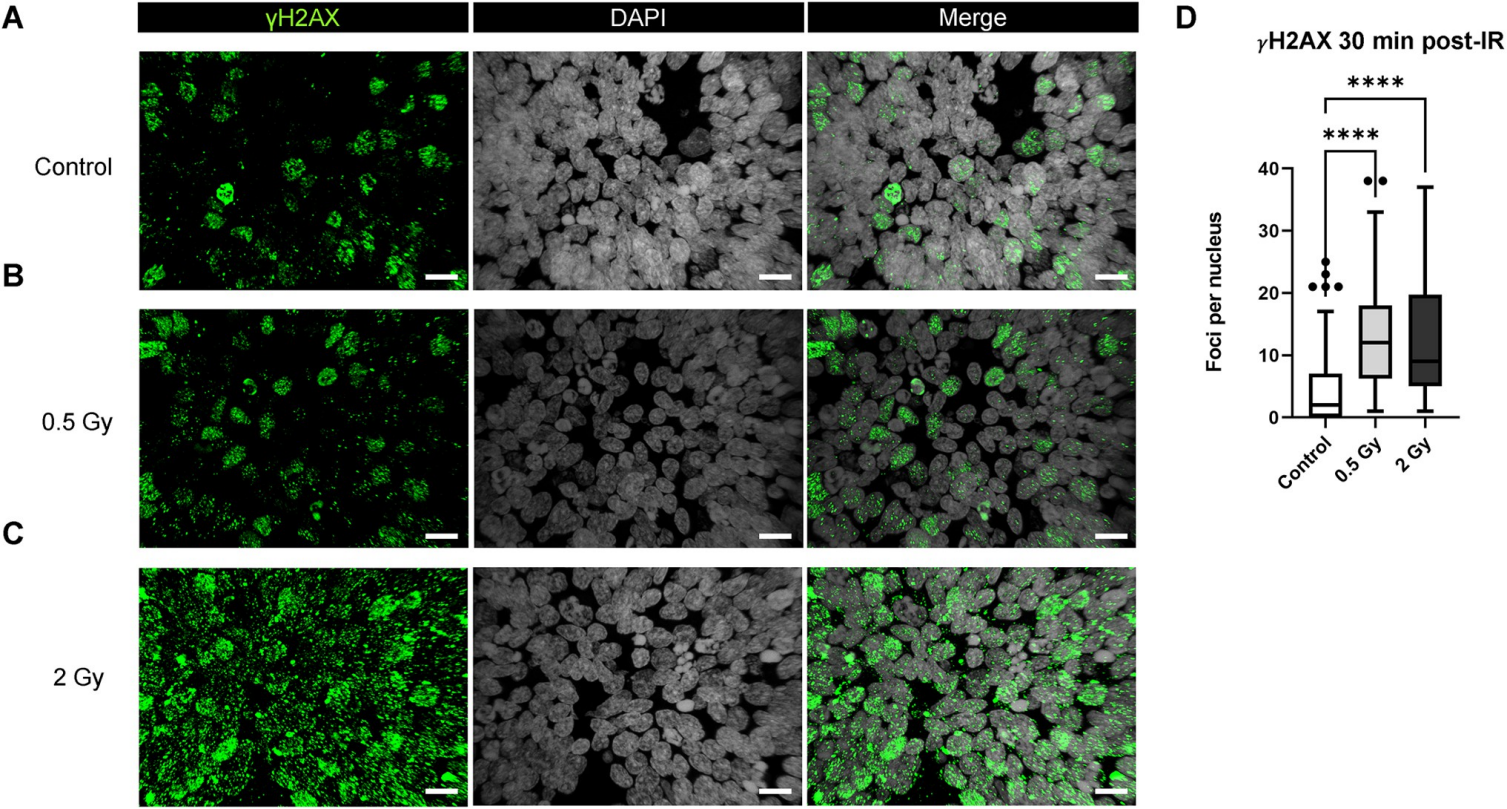
DNA damage in brain organoids 30 minutes post-irradiation. Immunostaining of DNA damage marker γH2AX in Day 70 WT83 C6 organoids 30 minutes after proton irradiation: (A) Control, (B) 0.5 Gy, and (C) 2 Gy. (D) Kruskal-Wallis test (P < 0.05) with Dunn’s multiple comparison test showing significant difference for control versus 0.5 Gy (P < 0.0001) and control versus 2 Gy (P < 0.0001) at 30 minutes (N = 3 organoids per group, n > 40 DAPI+ cell nuclei analyzed per group). The box-and-whisker plot shows the range and median of foci counts across regions of interest. Scale bars = 10 μm.

30 minutes after proton irradiation, the endogenous γH2AX expression was low in the unirradiated controls, with a median of 2 distinct foci per nucleus (Fig 3A). In comparison to controls, irradiated WT83 C6 organoids demonstrated higher expression of nuclear γH2AX. (Fig 3B and 3C). Additionally, immunostaining revealed larger γH2AX foci in the 2 Gy group (Fig 3C). The number of γH2AX foci significantly increased in the irradiated organoids, with a median of 12 distinct foci per nucleus in the 0.5 Gy group and 9 foci in the 2 Gy group (Fig 3D).

### Assessment of DNA damage in brain organoids 24 hours after exposure to 0.5 or 2 Gy of 250 MeV protons

**Fig 4:**
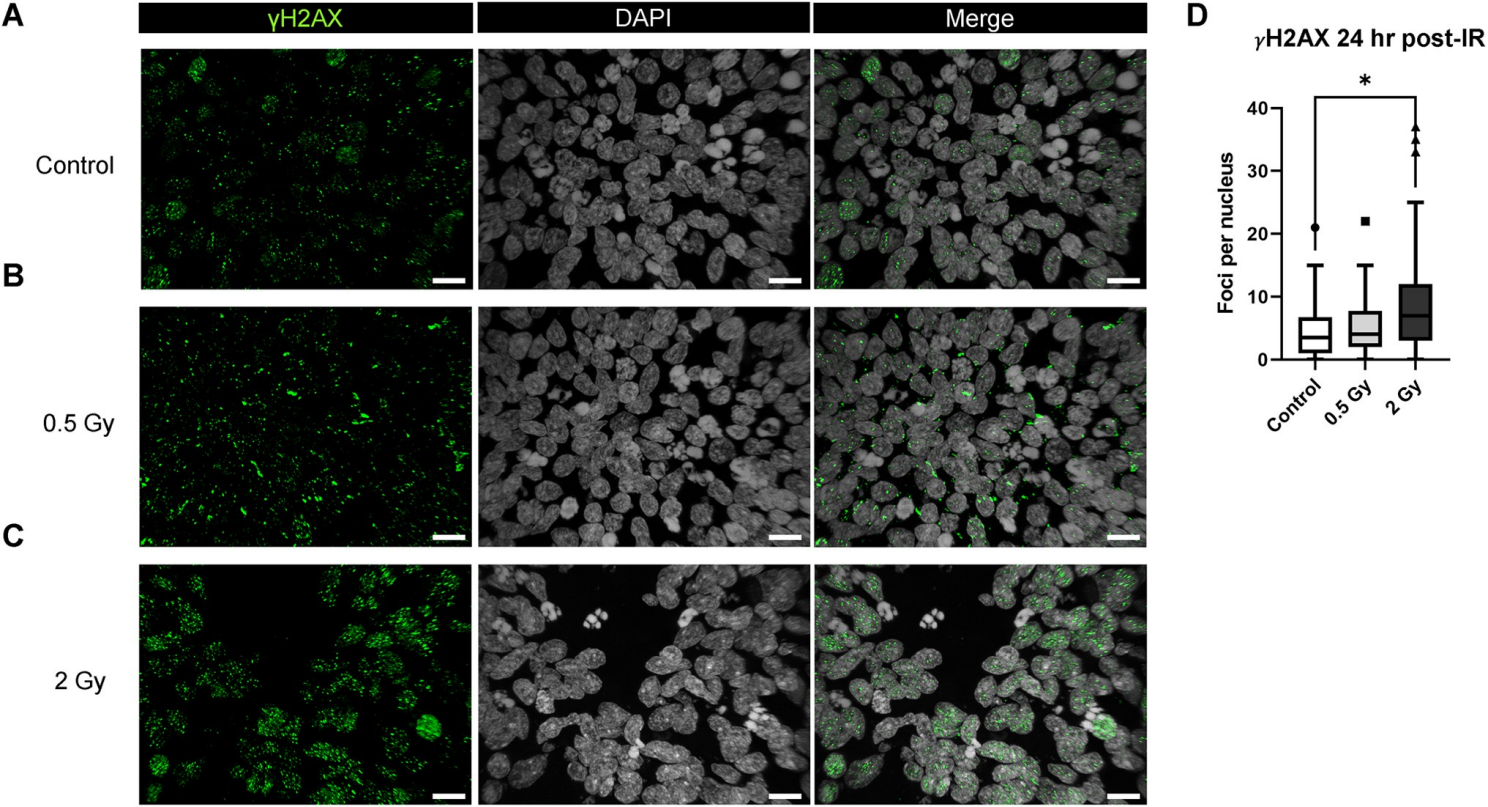
DNA damage in brain organoids 24 hours post-irradiation. Immunostaining of DNA damage marker γH2AX in Day 70 WT83 C6 organoids 24 hours after proton irradiation: (A) Control, (B) 0.5 Gy, and (C) 2 Gy. (D) Kruskal-Wallis test (P < 0.05) with Dunn’s multiple comparison test showing no change for control versus 0.5 Gy and significant difference for control versus 2 Gy (P < 0.05) at 24 hours (N = 3 organoids per group, n > 40 cell nuclei analyzed per group). The box-and-whisker plot shows the range and median of foci counts across regions of interest. Scale bars = 10 μm.

24 hours after exposure, the median number of foci per nucleus in the irradiated WT83 C6 organoids had decreased, relative to the 30-minute timepoint (Fig 4A–4C). The number of distinct γH2AX foci in the 2 Gy group (median = 7), but not the 0.5 Gy group (median = 4), remained significantly higher than the control group (median = 3.5) (Fig 4D).

### Assessment of DNA damage in brain organoids 48 hours after exposure to 2 Gy of 250 MeV protons

**Fig 5:**
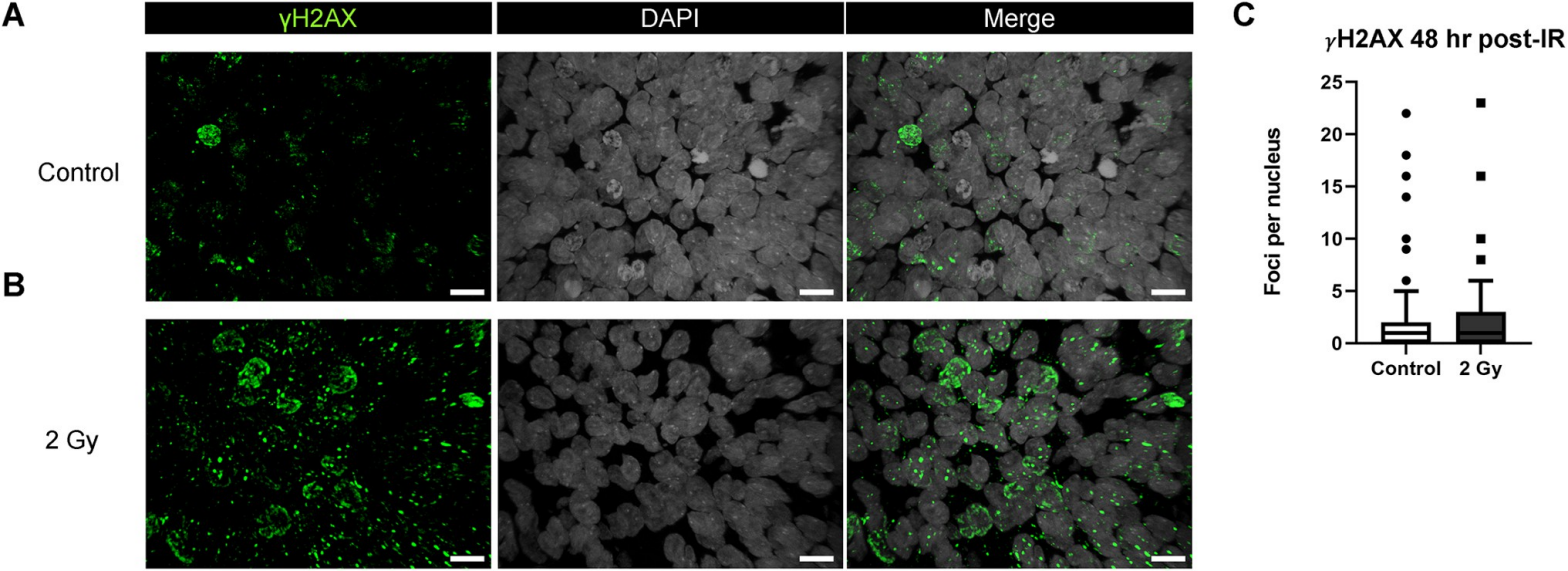
DNA damage in brain organoids 48 hours post-irradiation. Immunostaining of DNA damage marker γH2AX in Day 70 XB C2 organoids 48 hours after irradiation: (A) Control (B) 2 Gy. (C) Quantification of foci counts per cell nucleus with two-tailed Mann Whitney test (P < 0.05) showing no significant difference between unirradiated controls and irradiated organoids (N = 3 organoids per group, n > 40 cell nuclei analyzed per group). Box-and-whisker plot shows the range and median of foci counts across regions of interest. Scale bars = 10 μm.

**Fig 6:**
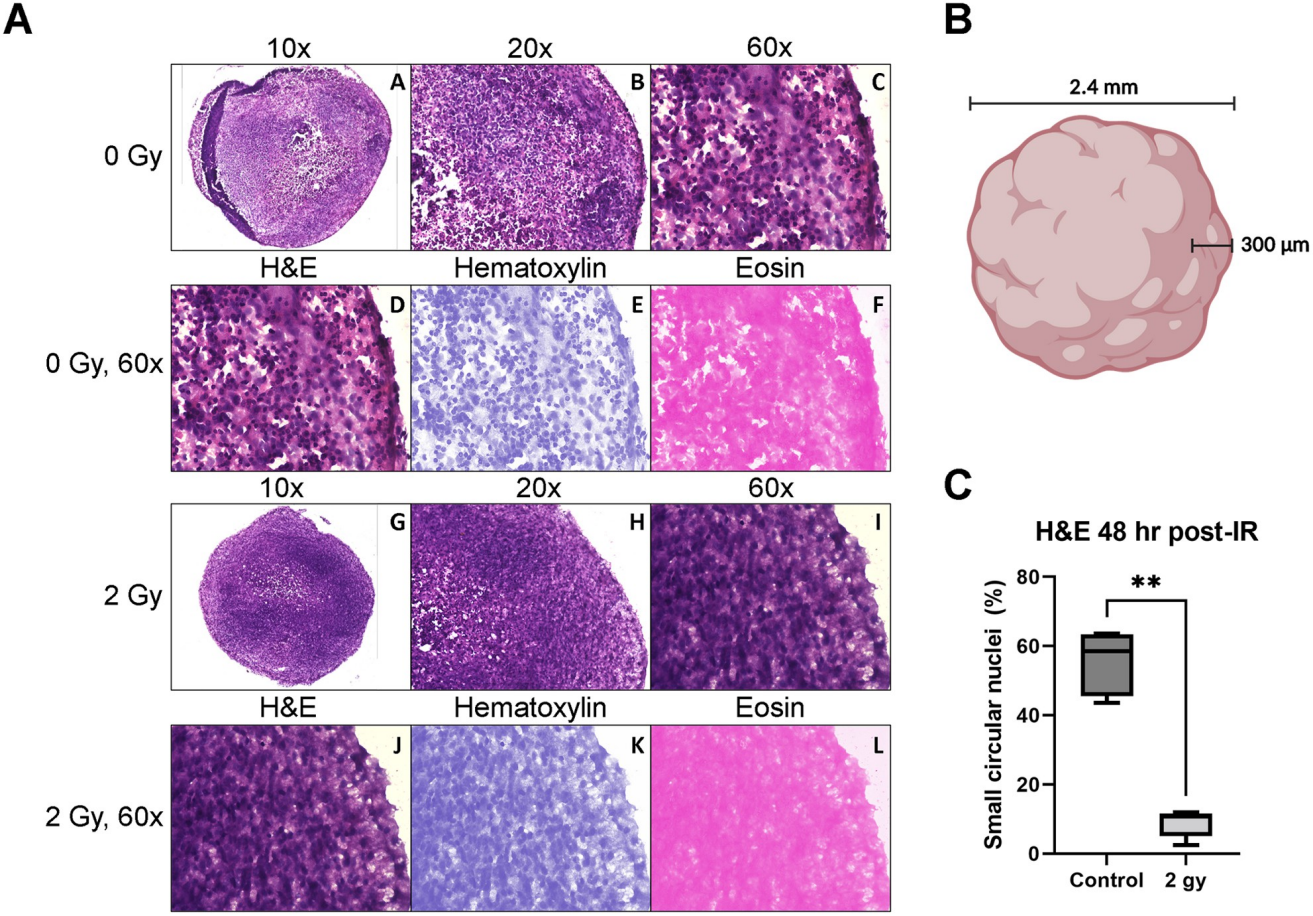
Hematoxylin and Eosin (H&E) staining of brain organoids. H&E staining of unirradiated control and 2 Gy irradiated Day 70 XB C2 organoids 48 hours after irradiation. The magnification used for microscopy is shown above each image (A-C, G-I). (A-C) Representative section of a control organoid shows outer regions with laminar cytoarchitecture and large ovoid cell nuclei surrounded by abundant small circular cell nuclei. (D-F) 60X magnified outer region of unirradiated control organoids with distributed nuclear and eosinophilic material separated using ImageJ Color Deconvolution. (G-I) Representative section of an irradiated organoid shows outer regions with large ovoid cell nuclei and few small circular nuclei. (J-L) 60X magnified outer region of 2 Gy irradiated organoids with distributed nuclear and eosinophilic material separated using ImageJ Color Deconvolution. (M) Illustration of an organoid showing the region of interest that was used to quantify small circular nuclei, within 300 μm of the outer surface. The illustration was created with BioRender.com. (N) Quantification of small circular nuclei with two-tailed Mann-Whitney U test (P < 0.05) showing a significant difference in counts between unirradiated controls and irradiated organoids (n = 5). Box-and-whisker plot shows the range and median of small circular nuclei proportions, relative to large ovoid nuclei, across regions of interest.

In brain organoids generated from the XB C2 cell line, by 48 hours, both the 2 Gy group and control group had a median of 1 distinct γH2AX focus per nucleus (Fig 5). H&E staining of control organoids showed a dense and uniform distribution of small, circular, hematoxylin-stained cell nuclei throughout the tissue alongside large, ovoid cell nuclei (Fig 6A–6F). However, there was a significant reduction of small, circular, hematoxylin-stained cell nuclei within 300 μm of the outer surface of the irradiated organoids (Fig 6G–6L and 6N). The eosinophilic material surrounding the cell nuclei was dense and uniform in both unirradiated controls and irradiated organoids.

### Quality control and DESeq2 analysis of bulk RNA-seq data 48 hours after exposure to 2 Gy of 250 MeV protons

**Fig 7:**
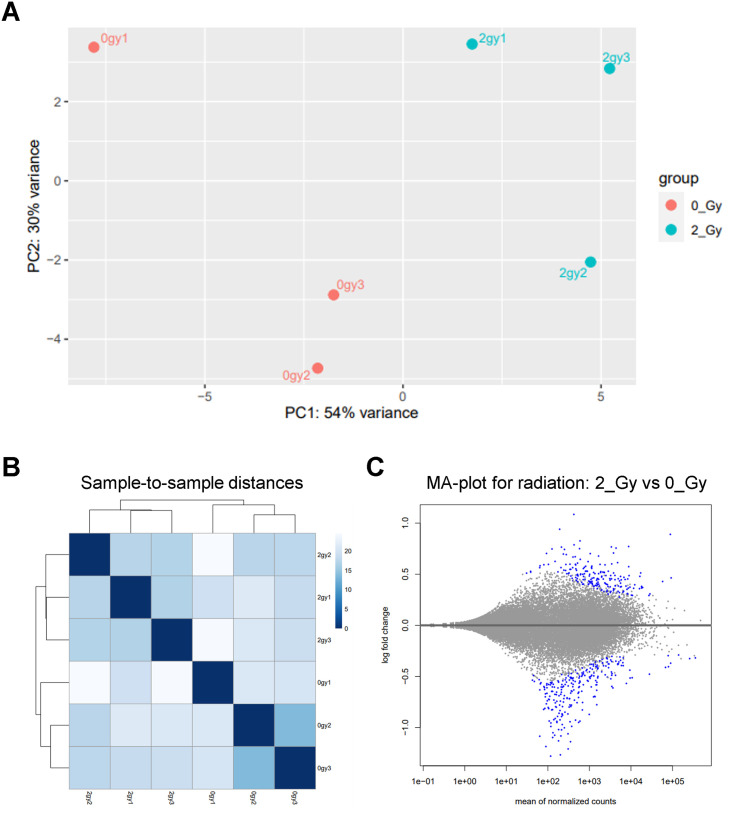
Gene expression changes of brain organoids 48 hours post-irradiation. (A) Principal Component Analysis (PCA) plot of the Control and 2 Gy irradiated XB C2 organoids. (B) Expression heat map showing sample-to-sample distances where darker blue represents greater similarity between samples. (C) MA plot showing the log (base 2) fold change (y-axis) versus the mean of normalized read counts (x-axis) using DESeq2. The alpha value for significance on the MA plot is set to 0.05, where each dot represents a single gene and significantly DEGs are colored blue.

The trimmed reads were of high quality, with all FASTQC quality scores exceeding 30 (S2 Fig). Principal Component Analysis (PCA) was used to analyze sample similarity based on normalized gene counts in XB C2 brain organoids. PC1 accounted for 54% of variance and it separates the irradiated group and control group; while PC2 accounted for 30% of the variance and was responsible for some variability within each set of replicates (Fig 7A). Sample-to-sample distances between replicates were low, indicating similar gene expression between replicates. Sample-to-sample distances between experimental groups were high, indicating differential gene expression following 2 Gy proton exposure (Fig 7B). In our analysis of differentially expressed genes (DEGs) between unirradiated controls and irradiated organoids, 445 genes showed a significant difference in expression 48 hours after irradiation (Fig 7C).

### Gene Set Enrichment Analysis (GSEA) of significant DEGs 48 hours after exposure to 2 Gy of 250 MeV protons

**Fig 8:**
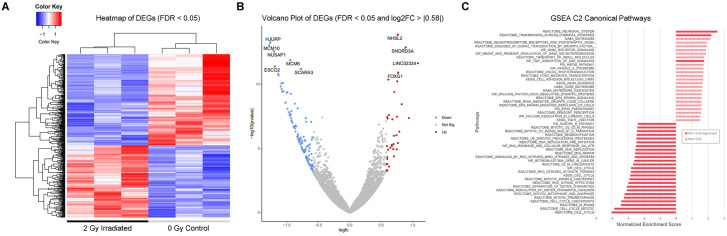
GSEA hallmark pathways and volcano plot. (A) Heatmap of significantly (FDR < 0.05) DEGs. The samples are clustered on the x-axis of the dendrogram based on their gene expression patterns. Columns 1–3 are the irradiated samples and columns 4–6 are the control samples. The dendrogram on the y-axis shows the relative expression patterns of individual genes across the samples. (B) Volcano plot showing DEGs with FDR < 0.05 and log2FC > |0.58| colored in red (upregulated) and blue (downregulated). The top 10 most significant DEGs are labeled. (C) Unsupervised gene set enrichment analysis results using MSigDB 7.5 C2 Canonical pathways for annotated genes, pre-ranked by fold change. On the graph, GSEA pathways are arranged according to their Normalized Enrichment Scores.

The initial output from DESeq2 contained a list of 64,838 gene IDs. After using the Ensembl Genome Reference Consortium Human Build 38 patch release 13 (build 105) to annotate the gene list and filtering out duplicates or entries that did not have an annotation, we pre-ranked the remaining 35,355 genes by fold change and performed GSEA of significant DEGs using Galaxy. In total, 445 genes (197 upregulated and 248 downregulated) were identified to be significantly differentially expressed, with adjusted p-value to account for false discovery rate (FDR/q-value < 0.05), using DESeq2 (Fig 8A). Applying a log2FC > |0.58| threshold, which corresponds to a fold change of 1.5, reduces the list of DEGs to 156 (35 upregulated and 121 downregulated) (Fig 8B). Among the top 10 most significantly DEGs, eight were protein coding genes (FOXG1, NHSL2, SCARA3, MCM6,​ ESCO2, NUSAP1, HJURP, MCM10), one was a small nucleolar RNA (SNORD3A), and one was a long noncoding RNA (LINC02334) (Fig 8B, Table 1). LINC02334 displayed the greatest upregulation (log2(FC) = 1.09), whereas MCM10 displayed the greatest downregulation (log2(FC) = -1.28) among all the significant DEGs. GSEA of the annotated DEGs using the Human MSigDB C2 sub-collection “Canonical pathways” identified which gene sets were most over-represented by the dataset. Reactome pathways “Neuronal System” and “Transmission Across Chemical Synapses” were the most positively enriched; pathways for cell cycle phases and checkpoints were the most negatively enriched (Fig 8C).

**Table 1:** Top 10 most significant DEGs.

Gene name	Function	FDR	log2(FC)
LINC02334	Unknown	5.03E-09	1.085255
SNORD3A	Unfolded protein response; implicated in prion disease progression and oxidative stress [49]	5.11E-10	0.890063
FOXG1	Expressed in the developing nervous system with a critical role in forebrain development [50]	1.87E-08	0.771255
NHSL2	Predicted to enable calcium ion binding activity; involved in cell differentiation [51]	2.10E-10	0.767008
SCARA3	Potential role in cellular stress response; encodes protein for scavenging harmful products of oxidation [52]	1.08E-08	-0.77671
MCM6	Minichromosome maintenance complex; DNA replication; cell cycle progression [53]	3.17E-09	-0.94979
ESCO2	Intermediate progenitor cell maintenance [54]	7.60E-09	-1.18577
NUSAP1	Encodes a microtubule-associated protein; spindle assembly, chromosome segregation, cytokinesis, and microtubule crosslinking [55]	7.95E-10	-1.2094
HJURP	Regulation of cellular senescence; cell proliferation [56]	4.52E-10	-1.26729
MCM10	Minichromosome maintenance complex; initiates DNA replication; protects against replication stress [57]	4.62E-10	-1.27977

Top 10 most significant DEGs and their functions, FDR values, and log2FC values are shown. These genes are sorted by their log2FC values from highest to lowest.

### Gene ontology (GO), KEGG pathway analysis, and identification of the most significant DEGs 48 hours after exposure to 2 Gy of 250 MeV protons

**Fig 9:**
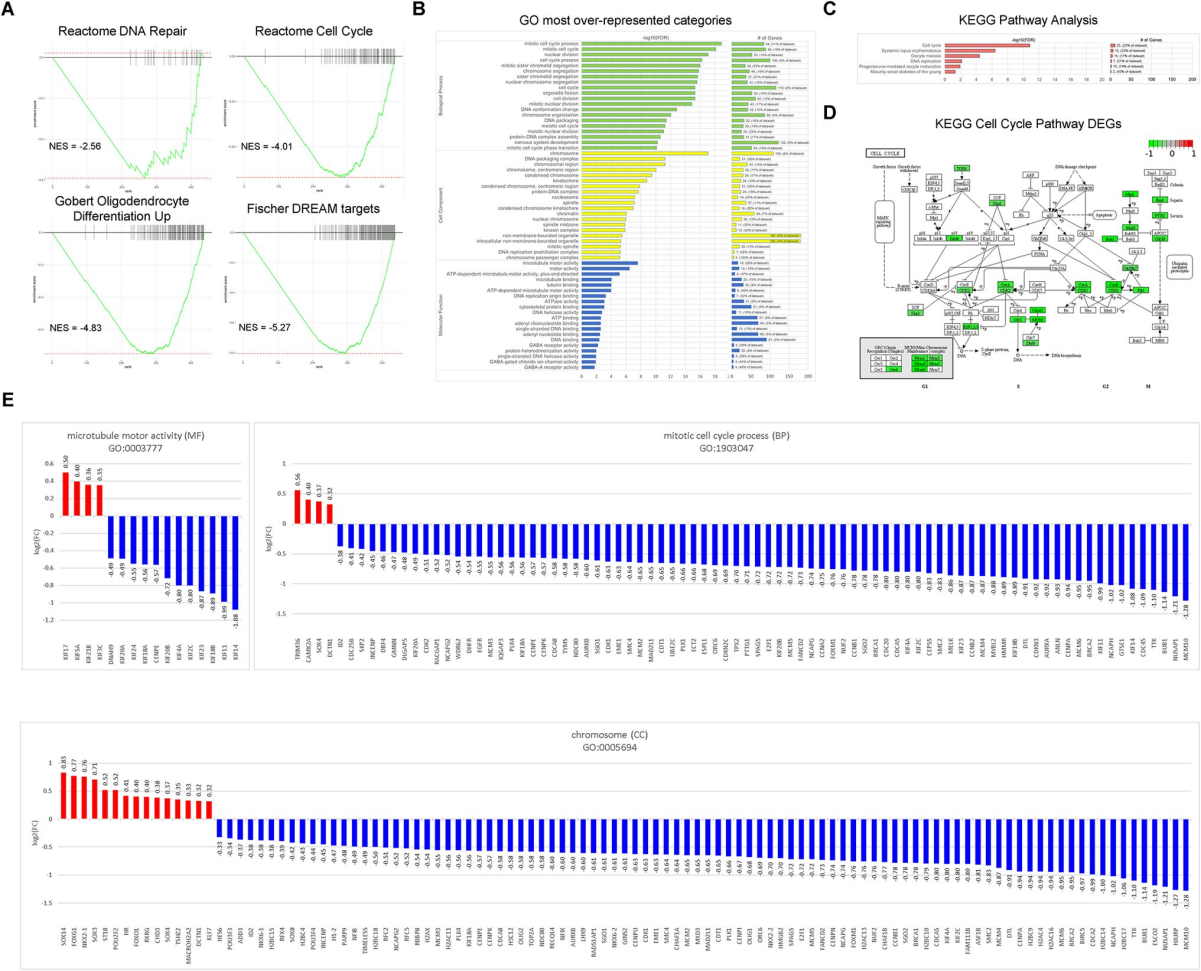
Top GO categories and KEGG pathways related to DEGs. (A) Enrichment plots of relevant pathways, showing running enrichment scores and positions of rank-ordered genes. (B) Gene ontology of the significant (FDR < 0.05) DEGs with the top 20 categories of each BP, CC, MF shown on the y-axis and fold change on the x-axis. The number of DEGs we identified in this category are shown in the graph to the right. (C) KEGG pathway analysis of the significant pathways with the largest number of DEGs. (D) KEGG visualization of the Cell Cycle pathway (hsa04110) adapted to show the replication fork in eukaryotes and overlaid with DEGs identified from the dataset. Red indicates upregulation, green indicates downregulation, and white indicates no change. (E) Expression ratios of DEGs in GO categories for the Molecular Function “microtubule motor activity”, Biological Process “mitotic cell cycle process”, and Cell Component “chromosome”, are shown sorted by their log2(FC) from highest to lowest. Upregulated genes are colored in red and downregulated genes are colored in blue.

Notably, we observed significant negative enrichment of the Reactome pathway “DNA Repair” (Fig 9A, Top Left); however, Reactome pathway “Cell Cycle” was the top over-represented (85 DEGs) and significantly negatively enriched (NES = -4.01) canonical pathway (Fig 9A, Top Right). We then analyzed our pre-ranked DEGs using all human gene sets in MSigDB and discovered that there was a significant negative enrichment of a gene set for oligodendrocyte differentiation (Fig 9A, Bottom Left). Of all human gene sets in MSigDB, our DEGs were most over-represented (137 DEGs) and significantly negatively enriched (NES = -5.27) in the gene set for Targets of the DREAM Complex (Fig 9A, Bottom Right). We then used GO analysis to identify canonical pathways that were enriched by DEGs in the dataset. The top ten most significantly over-represented categories were mitotic cell cycle process, mitotic cell cycle, nuclear division, chromosome, cell cycle process, mitotic sister chromatid segregation, chromosome segregation, sister chromatid segregation, nuclear chromosome segregation, and cell cycle (Fig 9B). KEGG Pathway Analysis revealed six pathways with the largest number of DEGs (FDR < 0.05): Cell cycle, systemic lupus erythematosus, oocyte meiosis, DNA replication, progesterone-mediated oocyte maturation, and maturity onset diabetes of the young (Fig 9C). Looking closer at the KEGG Cell Cycle pathway we see downregulation of protein-coding genes essential to the initiation of eukaryotic genome replication (Fig 9D). These genes encode proteins that form the pre-replication complex and the replication fork during the transition from G1 (cell growth) to S (DNA synthesis). Genes encoding proteins that are active during S were also downregulated (Fig 9D, S1 Data). The results of our study clearly demonstrated that DNA damage and the subsequent repair are major responses of the cell to IR. Among the GO categories, there were 16 genes expressed in “microtubule motor activity” (Molecular Function, MF), 84 genes expressed in the “mitotic cell cycle process” (Biological Process, BP), and 109 genes expressed in “chromosome” (Cell Component, CC) (Fig 9E).

Notably, GO revealed significant (FDR < 0.05) overrepresentation of genes in cellular response to DNA damage stimulus, DNA repair, central nervous system development, cell differentiation, glial cell fate commitment, neurogenesis, gliogenesis, oligodendrocyte differentiation, neuron projection morphogenesis, and regeneration. IR significantly affects mitochondrial function, and we found that mitochondrial genes were downregulated in this study (S1 Table). Furthermore, we were interested to see if cell-specific genes of interest from our dataset were changed after irradiation and found that expression of NEUN/RBFOX3 (RNA binding fox-1 homolog 3, log2(FC) = 0.61, FDR = 0.0013), was significantly upregulated, along with neuron-neuron (NECTIN3, L1CAM, CADM1, NRCAM) and neuron-myelinated cell (NRCAM, NFASC, VCAN) adhesion molecules. Oligodendrocyte-associated genes OLIG1 (oligodendrocyte transcription factor 1, log2(FC) = -0.68, FDR = 0.0050), OLIG2 (oligodendrocyte transcription factor 2, log2(FC) = -0.58, FDR = 0.017), OMG (oligodendrocyte myelin glycoprotein, log2(FC) = -0.51, FDR = 0.040) and MBP (myelin basic protein, log2(FC) -0.54, FDR = 0.025) were significantly downregulated by 48 hours. There were no significant DEGs related to astrocyte lineage (S1 Data).

### Ingenuity pathway analysis of DEGs 48 hours after exposure to 2 Gy of 250 MeV protons

**Fig 10:**
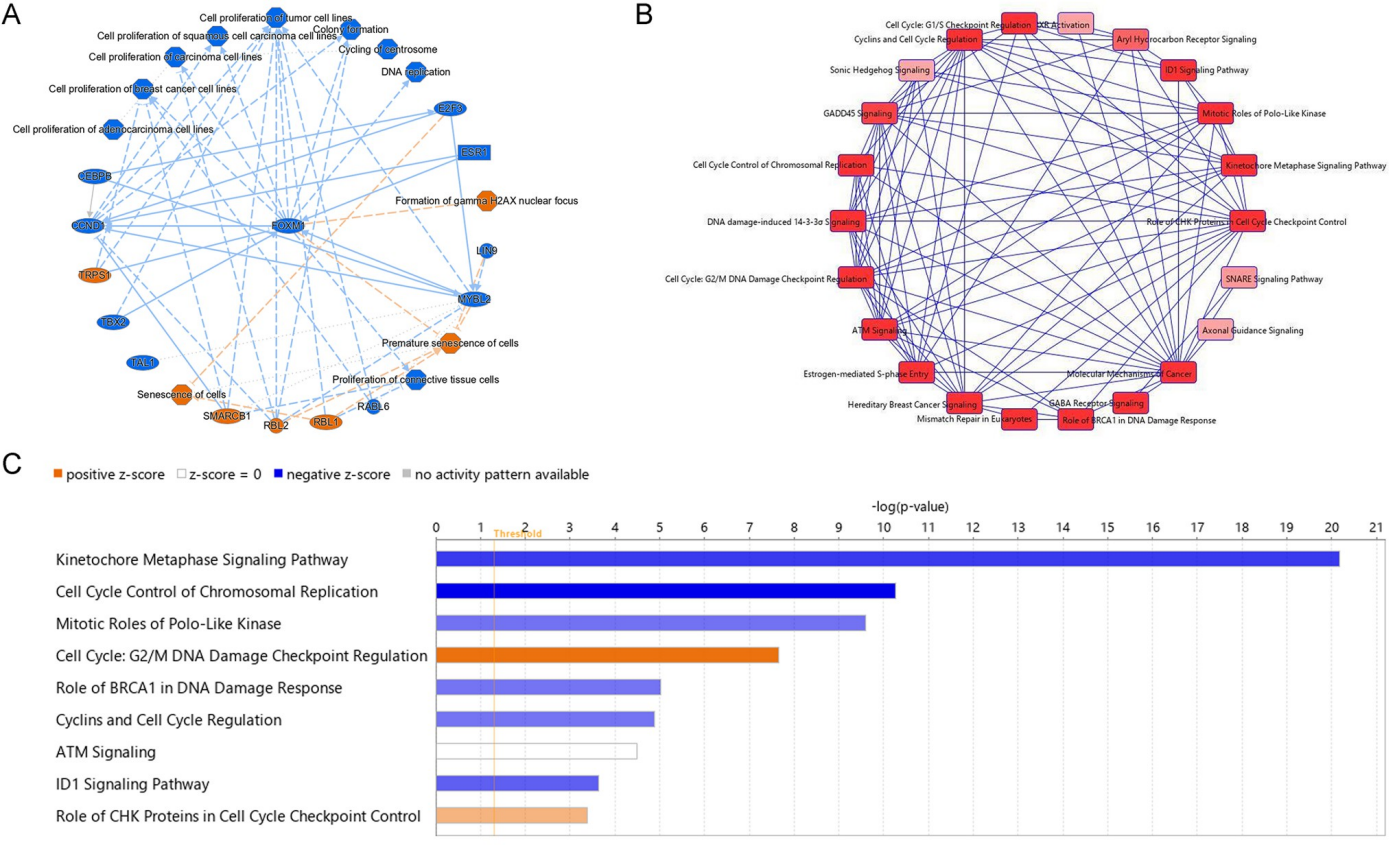
Ingenuity pathway analysis of DEGs. (A) A graphical summary of significant DEGs with p < 0.05 and log2(FC) > |0.58| was generated using Ingenuity pathway analysis (IPA) to identify the major biological themes in the dataset. (B) IPA representation of overlapping canonical pathways with lines connecting pathways that share common genes. (C) Graph of canonical pathways with -log(p-value) > 1.3 (p-value < 0.05) sorted from most to least significant.

Ingenuity Pathway Analysis (IPA) was used to identify radiation induced changes in pathways relevant to cell cycle progression, genomic stability, and cell senescence (Fig 10A). We identified canonical pathways associated with the DEGs in our dataset using IPA, which were mostly related to cell cycle progression, DNA damage response, and synaptic activity (Fig 10B). The Kinetochore Metaphase Signaling Pathway was identified by IPA to be the most represented pathway in our dataset (Fig 10C). Our results are consistent with indications of organismal injury and tumorigenesis via IR-induced DNA damage and chromosome breaks.

## Discussion

Due to the increasing likelihood of exposure to low doses of ionizing radiation (IR) in clinical and occupational settings, it is becoming increasingly important to characterize the radiosensitivity of different cell types, particularly the subclinical responses of human brain tissue. The National Academies of Sciences, Engineering, and Medicine recently released a report highlighting the urgency of making advances in radiobiological research, which brought up an urgent need to use brain organoids in IR research [58]. As we have shown, measures of radiosensitivity (such as differential cell viability and γH2AX expression) vary with dose and time after exposure. This raises great concerns of using traditional approaches to quantitate radiosensitivity. In this study, we demonstrate that RNA-Seq is a powerful tool to identify novel biomarkers in brain organoids after exposure to IR. The 70-day timepoint for organoids was selected based on previous literature, which demonstrated that mature neurons, astrocytes, myelinating oligodendrocytes, and persistence of neural stem cells could be detected [46, 59]. Based on IF staining and RNA-Seq, organoids we cultured for 70 days contained both neural stem cells and mature/differentiated cells, but no microglia. Microglia, the resident immune cells of the brain, are also known to play a significant role in the human brain response to IR. Low dose IR elicits a phenotypic change in microglia from canonically pro-inflammatory to canonically anti-inflammatory, allowing them to carry out neuroprotective function and restore the brain environment through neurotrophic factors, whereas high dose IR promotes the pro-inflammatory phenotype [60, 61]. Our model can be modified as needed to contain microglia, providing insight into the essential role of microglia in regulating neuroinflammation [43, 46]. For characterization of organoids 30 minutes and 24 hours after irradiation, we used three technical replicates of embedded organoids for each dose and timepoint, with at least three organoids per replicate. For characterization of organoids 48 hours after irradiation, we divided organoids between IF staining and RNA sequencing, maintaining three technical replicates (per experimental group) of each assay, with at least three organoids per replicate.

The doses used in our study doses are relevant for fractionated treatment of primary brain tumors, as well as for estimating health risks for astronauts exposed to proton radiation during long-duration space missions [62–64]. In clinical settings, IR is used alone, or in combination with chemotherapy and surgery, to reduce brain tumors [65]. Most patients worldwide are treated with photon beams (energy ranging from 4–18 megavolt) and less than 1% are treated with protons (energy ranging from 70–250 MeV) and heavier ions [66]. Using protons, radiation oncologists can achieve a high therapeutic ratio, where the dose is concentrated in the target volume and minimized in the surrounding healthy tissue [65]. Radiotherapy patients typically receive fractionated doses of 1.8–2 Gy up to ~60 Gy over a period of weeks [67]. Though there are many strategies to mitigate exposure risks in a clinical setting, protecting astronauts from space radiation is an even greater challenge [27]. A 2 Gy dose of ionizing radiation that would be considered “low-moderate” in radiotherapy is considered a high dose in the context of spaceflight [27, 64]. Astronauts can expect to be exposed to a cumulative dose of over 0.2 Gy per year from Galactic Cosmic Rays, composed of protons, helium nuclei, and high charge and energy (HZE) nuclei (energy ranging from ~10–10,000 MeV/n) [27]. However, because there is much less shielding when performing extravehicular activities, during a solar particle event an astronaut could absorb cumulative doses (energy ranging from ~10 to over 100 MeV) that exceed NASA’s 30-day deep tissue dose limit of 0.25 Gy [27, 68]. With minimal shielding protection from these high energy particles and the inability to rapidly receive “standard of care” treatment, IR exposure can significantly affect crew health and the mission’s objectives [68]. We aim to develop a model that will enable us to determine how proton radiation affects the health of the human brain during clinical exposures as well as during space travel.

IF staining revealed no significant change in NeuN+ cell counts or the number of GFAP+ cell processes at 30 minutes or 24 hours after irradiation with either dose. RNA-Seq demonstrated that there was no statistically significant difference in GFAP expression between the control and irradiated organoids, but RBFOX3/NEUN expression increased significantly 48 hours after irradiation with 2 Gy protons. The increase in NEUN expression and concurrent downregulation of cell cycle genes suggest that neural stem/progenitor cells have switched from proliferation to differentiation/neurogenesis at 48 hours after IR exposure. Analysis of MBP+ cell counts revealed a significant difference in value between groups using the Kruskal-Wallis test (p = 0.0250), but the strong trend showing a difference between the 0.5 and 2 Gy irradiated organoids did not reach significance by Dunn’s multiple comparisons test (p = 0.0512). However, RNA sequencing supports these findings, showing a significantly decreased expression of oligodendrocyte associated genes OLIG1, OLIG2, OMG, and MBP 48 hours after organoids were irradiated with a 2 Gy dose. The results suggest that mature neurons and astrocytes are acutely resistant to radiation-induced cell death at low doses, whereas oligodendrocytes are acutely sensitive.

The prefrontal cortex and neurogenic niches of the human brain (subgranular zone of the hippocampal dentate gyrus and subventricular zone of the lateral ventricle) are reported to be the most sensitive to irradiation [69, 70]. Low-moderate dose IR is known to alter radiosensitivity, proliferation, apoptotic rate, and DNA damage in neural stem cells, which are found in humans in the developing brain and neurogenic niches of the adult brain [71]. Surprisingly, we did not detect statistically significant changes to the gene expression of neural stem cells markers including SOX2, NESTIN, and PAX6 48 hours after 2 Gy irradiation. After exposure to doses ≤ 2 Gy of IR, cultured neural stem cells and mice brains exhibit acute dose-dependent increases in oxidative stress and neuroinflammation that become persistent, thus contributing to cognitive impairment [32, 72–74]. Previous studies show increased apoptosis of proliferating cells and immature neurons in the rodent hippocampus, which peaked 3–12 hours and then returned to near control levels by 48 hours after exposure to ≥ 2 Gy X-rays [75, 76]. The total number of BrdU-positive cells decreased 24 hours after ≥ 2 Gy X-rays in rats [75]. Another study reports that in the developing mouse brain, 2 Gy irradiation induces significant apoptosis of neural precursors and sparing of neurons by 24 hours; γH2AX+ foci counts are similar between neural precursors and neurons at 1 hour and at 24 hours, but with slower rate of DNA repair in neural precursors [77]. As stated above, it is important to distinguish between radiobiological effects following exposure to different types of IR, since each elicits different responses [78]. Others have shown that there was no change in neurogenesis, based on counts of BrdU+/NEUN+ cells, in WT mice 1 month after 2 Gy gamma irradiation. However, they did show a significant increase in NEUN+ cells using a radioadaptive dosing regimen of 10 cGy followed by 2 Gy [79]. Despite the significant changes in populations of neural precursor cells, the study did not observe apoptosis in fully differentiated neurons after doses up to 30 Gy [75].

The presence of γH2AX+ foci within organoids after exposure to a 0.5 or 2 Gy dose of 250 MeV protons indicates strand break-induced activation of DNA damage response proteins 30 minutes after irradiation. After 24 hours, we observed a sustained increased in γH2AX+ foci expression in 2 Gy irradiated organoids, whereas signal expression in 0.5 Gy irradiated organoids resembled the control group. Accordingly, previous studies show that exposure to increasing doses of IR correlates with more complex DNA damage and delayed repair [80]. After 48 hours, γH2AX expression was similar between the 2 Gy irradiated organoids and the control group. These results are consistent with previous findings showing that radiation-induced DNA damage elicits a peak response of DNA repair mechanisms within 30 minutes of exposure, with surviving cells able to repair most DNA strand breaks within 24 to 48 hours after exposure [81–84].

In the brains of mice exposed to 2 Gy protons, numerous genes are modulated, including those related to increasing oxidative stress and reducing antioxidant defense [85]. The observation that lipid peroxidation is a primary agent of IR-induced oxidative stress, which provides a possible explanation for the radiosensitivity of lipid-rich myelinating oligodendrocytes.

Furthermore, the integrity of mitochondrial function is critical for ATP production and regulating oxidative stress. Neural precursor cells exposed to the Bragg Peak of 250 MeV protons at doses 1, 2, 5, or 10 Gy demonstrated increased levels of reactive oxygen species that were not associated with altered mitochondrial functions and content [86]. In this study, we identified significant downregulation of genes which encode proteins that are critical subunits of complexes in the electron transport chain of mitochondrial oxidative phosphorylation. Unresolved mitochondrial dysfunction contributes to neurodegenerative diseases such as Leigh’s syndrome, though no correlation with early-life radiation exposure has been identified [87–89]. However, individuals with inherited genetic disorders including Leigh’s syndrome and ataxia telangiectasia are known to be hypersensitive to IR due to an inability to repair or respond to DNA damage [90, 91]https://paperpile.com/c/LwjXT6/w5u9+SKWY. Genes related to cell cycle progression and DNA damage repair were the most significantly differentially expressed in the dataset, as shown by downregulation of protein-coding genes for DNA replication with MCM10 showing the greatest downregulation in the dataset. Radiation-induced downregulation of MCM10 in cells has been shown to occur within hours of exposure to 50 J/m^2^ UV radiation or 2 Gy photons [92, 93]. However, to our knowledge, we are the first to report radiation-induced downregulation of MCM10 using organoids. In our study, 16 long non-coding RNAs were significantly differentially expressed in our dataset: ENSG00000274317 (LINC02334), ENSG00000186960 (LINC01551), ENSG00000125462 (MIR9-1HG), ENSG00000231764 (DLX6-AS1), ENSG00000248540 (RP11-247C2.2/LOC283731), ENSG00000245526 (LINC00461), ENSG00000260412 (RP11-438B23.2/LINGO2), ENSG00000229415 (SFTA3), ENSG00000246731 (MGC16275), ENSG00000260918 (RP11-731J8.2/GABRB1), ENSG00000250742 (LINC02381), ENSG00000162913 (OBSCN-AS1), ENSG00000281327 (LINC01338), ENSG00000259439 (LINC01833), ENSG00000260807 (CEROX1),. ENSG00000258498 (DIO3OS). Among these genes, as well as all the DEGs in the study, LINC02334 had the greatest fold change. This long non-coding RNA has only previously been associated with the brain for risk of schizophrenia, neurofibrillary tangles, and PHF-tau in Genome Wide Association Studies [94]. Thus, to our knowledge, we are first to report that these long non-coding RNAs, particularly LINC02334 are associated with radiation-induced cell responses and could serve in future studies as biomarkers for radiation exposure or neurodegeneration/dementia. Comparing these results to existing literature, the downregulation of DNA damage repair genes by 48 hours provide further evidence that low-moderate dose proton irradiation can inhibit DNA damage repair [95]. A recent study found identified radiation type- and dose-specific transcriptional responses across healthy and diseased mammalian tissues reported that low doses up to 0.5 Gy are related with cytokine cascades, while higher doses with ROS metabolism [96]. This study reported significantly enriched GO pathways associated with 0.6–2 Gy doses of IR that were also significantly enriched in our study: negative regulation of mitotic cell cycle (GO:0045930), negative regulation of cell cycle process (GO:0010948), mitotic cell cycle phase transition (GO:0044772), organelle fission (GO:0048385) [96]. In addition, their study reported that transcriptional responses such as immunomodulation, inflammation, oxidative stress responses, and cell death were different from those caused by lower doses or less energetic radiation [96]. We also identified similar changes in gene expression to what was reported in a study comparing irradiated (≤ 2 Gy) mice to patient’s with Alzheimer’s disease and identified significant modulation of genes for synaptic transmission, GTPase activity, MAPK signaling, Ephrin signaling, serine metabolism, kinesins, solute-carriers, GABA signaling, glutamate signaling, cytoskeletal reorganization, potassium channels, and ubiquitination [24, 97].

In a recent study, 15- and 60-day old forebrain (subventricular zone) organoids were exposed to 2 Gy X-rays. In the irradiated 15-day old organoids, the highest number of γH2AX+ and 53BP1+ foci at DNA double strand breaks were observed 1 hour after irradiation which were mostly repaired after 6 hours and almost completely repaired by 18–24 hours. In the 60 day old organoids, a similar number of foci were observed at 6, 18, and 24 hours after radiation and differences in repair rates between SOX2+ progenitors and more mature CTIP+ neuronal cells were also observed, with a more significant reduction in the number of γH2AX foci in SOX2+ cells [98]. In a similar study, 2–3 month-old organoids were exposed to 2 Gy X-rays and γH2AX+ and 53BP1+ foci at DNA double strand breaks were quantified. Authors observed a significant increase in the expression of these markers 30 minutes after irradiation with repair at 18 and 72 hours, aside from persistence of larger foci. However, in contrast to the work of Das et al. described above, the second study observed more persistent γH2AX foci in SOX2+ cells relative to SOX2- cells [99]. Compared to these studies, we observed a higher number of γH2AX foci 30 minutes after 2 Gy proton irradiation and similar numbers of foci at 24 hours, while quantification of cell-type specific differences in foci counts is ongoing. Additionally, the organoids in our study were more mature (70 days old) and contained MBP+ myelinating oligodendrocytes. As we and others have shown, brain organoids are useful models because they demonstrate the cell diversity and even rudimentary cytoarchitecture of the human brain.

Radiation exposure can have wide-ranging effects on biological tissue, many of which are not yet fully understood or elucidated. Consequently, the dose and duration of exposure to radiation using brain organoids may be limited by a variety of factors, i.e., the lack of a vascular system and immune components [61]. For example, the circulatory system plays a vital role in eliminating radiation damage from exposed tissues, meaning post-irradiation release of stress factors and signaling molecules may not be properly addressed and cleared. Moreover, brain organoids may lack the multi-organ dependent cell diversity and migratory activity relevant to the response to radiation-induced damage that occur within the human brain. As organoids grow, their cores can become necrotic due to poor nutrient diffusion and gas exchange, along with inadequate waste elimination [100, 101]. Furthermore, chronic effects of radiation exposure over a long period of time and continuous dose exposures experienced by some populations (e.g. astronauts) are both logistically and financially challenging to model using brain organoids. However, ongoing studies, e.g. organ-on-a-chip systems, vascularization, and host-integration approaches, offer a solution to improve organoid maturation and nutrient supply [102–104]. As these methods improve, we expect that future organoid models will provide a more accurate assessment of the risks of human brain exposure to IR, including the effects on specific brain regions. In the future, studies may investigate “bystander” effects of radiation, as they can alter gene expression, affect dendritic morphology, and lead to cognitive deficits in animals [70]. Furthermore, subpopulations of brain cells can be interrogated using more specific markers to further differentiate the population-specific consequences of exposure. In the future, brain organoids may be useful for evaluating the biological effects of various radiotherapy techniques to improve patients’ outcomes. Single-cell sequencing and spatial transcriptomics can be used to study these responses in the spatial and temporal context more closely, i.e. cellular interaction and communication. Future studies would also benefit from a greater sample size of organoids to be used in histology and RNA-Seq. The organoid model we have presented can be adapted to study the dose-specific effects of protons on cell population ratios, neural cell axon and dendrite projection, DNA damage, and gene expression. Moving forward, we plan to use this model to further characterize cell-specific measures of radiosensitivity such as cell proliferation, differentiation, DNA damage repair rates, morphological changes, cell migration, and apoptosis using additional radiation types, post-exposure timepoints, and radiation doses/dose-rates.

## Conclusion

In conclusion, we used immunohistochemical staining and RNA-Seq of brain organoids to elucidate cell- and tissue-specific changes after exposure to 0.5 and 2 Gy doses of proton radiation. Our results demonstrate that brain organoids can be used to reliably monitor acute changes in cell state and function following exposure to IR, with the potential to monitor these effects long-term. These findings are important to evaluate the potential health risks to individual human brain after IR-exposure as well as to predict the long-term risks of such exposure.

## Materials and methods

### Human induced pluripotent stem cell (hiPSC) culture

Two distinct hiPSC lines (WT83 C6 & XB C2) were used to generate organoids in this study (S2 Table). The methods used to generate and characterize these lines have been described previously [105, 106]. The WT83 C6 line was cultured in mTeSR1 (StemCell Technologies #85851) on 60 mm dishes coated with Matrigel (Corning #354234) and manually passaged/expanded using a P1000 pipette. The XB C2 line was cultured in mTeSR Plus (StemCell Technologies #100–0276) on 60 mm dishes coated with Matrigel (Corning #354234) and passaged/expanded using ReLeSR (StemCell Technologies, #100–0484). Donated fibroblasts were obtained via skin biopsies from healthy patients after informed consent was appropriately given under protocols approved by the University of California, San Diego Institutional Review Board (#141223ZF). This study was approved and performed following the Loma Linda University Institutional Review Boards (IRB) and Institutional Stem Cell Research Oversight Committees guidelines and regulations.

### Generation of 3-D brain organoids

To generate brain organoids, hiPSC cultures were dissociated into a single cell suspension using Accutase (Innovative Cell Technologies, Inc., #AT-104) for 20 minutes at 37 °C, diluted with PBS, transferred to a 15 mL conical tube, and then centrifuged for 5 minutes at 300 x g. The supernatant was removed and the hiPSCs were resuspended in warm mTeSR Plus with 10 μM Rock inhibitor (StemCell Technologies #72304) and counted on an automated cell counter (Bio-Rad, #1450102). Approximately 3 × 10^6^ hiPSCs were seeded per well of a 24-well AggreWell-800 plate (StemCell Technologies, #34815) which was centrifuged at 100 x g for 3 minutes to form spheroid aggregates (Day 0). After 24–48 hours, the spheroids were dislodged from the microwells and transferred to an Ultra-Low Attachment 6-well plate (Costar, #3471) using a wide bore P1000 pipette tip. The spheroids were maintained in suspension culture on an orbital shaker (~95 rpm) and guided via directed differentiation into brain organoids using previously described methods (S2 Table) [46].

### Radiation exposure

At Day 70 of suspension culture, the organoids were transferred to the LLU proton facility and irradiated with a single 0 (Control), 0.5, or 2 Gy dose of 250 MeV protons at a dose rate of 85.2 cGy/min. The cultures were kept at 37 °C in an insulated unit during transportation. During the irradiation process cultures were at room temperature (RT) for less than 5 minutes. Immediately afterwards, they were returned to the 37 °C incubator. Organoid irradiation utilizing a therapeutic proton beam was completed at the James M. Slater Proton Treatment and Research Center, Loma Linda California. For these irradiations 250 MeV protons were modulated to generate a 5.0 cm wide spread-out Bragg peak (SOBP). The cells were located at a water equivalent depth of 29.6 cm, specified using water-equivalent plastic blocks (CIRS, Norfolk, VA), which placed the cells in the uniform dose SOBP region of the proton dose profile. The proton field size employed for the irradiation of the organoids was circular with an 18 cm diameter. Protons were delivered from our synchrotron accelerator in a pulsed fashion, with a pulse duration of 0.125 seconds and a duty cycle of 2.2 seconds. Dose profiles were evaluated using Markus Parallel plate ionization chamber (s/n 23343–1771; PTW, Freiburg, Germany) and Keithley electrometer (Keithley, Cleveland, Ohio). The beam yielded a uniform dose distribution across the irradiated organoids at the 90% isodose line.

### Organoid tissue and media collection

For the experiments involving the WT83 C6 cell line, Day 70 organoids were collected at 30 minutes and 24 hours post-IR exposure, immediately fixed in 4% paraformaldehyde (PFA), and then stored overnight at 4°C. The next day, the 4% PFA was replaced with a 30% sucrose phosphate-buffered saline (PBS) solution and then stored overnight at 4°C for dehydration and cryoprotection. The following day, the organoids were transferred to Peel-A-Way embedding molds (Epredia, #12–20), embedded in Optimal Cutting Temperature (OCT) (Sakura Finetek™ #4583) solution on dry ice, and then stored long-term at -80°C. Organoids were later cryosectioned at 20 μm using a Leica CM1850 cryostat onto Superfrost Plus microscope slides (Fisherbrand #12-550-15). Each section contained 2–4 organoids and there were three sections per slide. The slides were dried and stored at -20 °C in a slide box prior to histology. Aside from collecting the organoids at 48 hours post-IR exposure, all steps were identical for the Day 70 organoids generated from the XB C2 cell line.

### Tissue preparation for immunohistochemistry and histology

Slides were thawed at RT and then submerged in 100% methanol for 5 minutes to permeabilize the tissue. Then, the sections were washed in PBS-Glycine (2.25 g/L) on The Belly Dancer Shaker (speed 1) (Alkali Scientific, #BD01) three times for 5 minutes each wash. Sections were permeabilized and blocked in PBS-Glycine containing 0.5% Triton and 3% Normal Goat Serum at RT for 30 minutes. Afterwards, slides were washed three times (5 minutes each wash) in PBS-Glycine and then incubated overnight with primary antibodies in PBS-Glycine containing 0.3% Triton X-100 and 5% NGS. The following primary antibodies were used in the study: γH2AX (1:250, Abcam #ab81299) and 53BP1 (1:250, Abcam #ab175933) for DNA damage; NeuN (Abcam #ab104224), beta-Tubulin III (1:500, Abcam #ab7751), Neurofilament Heavy (1:500, Abcam #ab4680), and MAP2 (1:250, Abcam #ab5392) for neurons; NESTIN and SOX2 for neural stem cells (1:250, Abcam #ab79351); Olig2 (ThermoFisher #MA5- 15810) and MBP (ThermoFisher #PA-10008) for oligodendrocytes; GFAP(1:500, Abcam #ab4674) for astrocytes. The following steps were carried out in low light conditions. Slides were washed five times in PBS-Glycine (5 minutes each) on a rotating shaker and then incubated with secondary antibodies in PBS-Glycine containing 0.3% Triton X-100 and 5% NGS at RT for 1.5 hours. The following secondary antibodies were used: Rabbit 488 (1:500, Invitrogen #A-11070), Mouse 555 (1:500, Invitrogen #A-31570) and Chicken 647 (1:500, Invitrogen #A-21449). Afterwards, the slides were washed three times in PBS-Glycine (5 minutes each). Slides were incubated for 5 minutes with ReadyProbes^™^ Tissue Autofluorescence reagent at RT and then washed three times in PBS-Glycine (5 minutes each). After the final wash, the cell nuclei were counterstained using Prolong Gold Antifade Mountant with DAPI (Invitrogen #P36931) and then slides were mounted with coverslips. To view the tissue morphology, cryosections were stained with the Hematoxylin & Eosin stain kit (Abcam #ab245880) according to the manufacturer’s protocol.

### Confocal and brightfield microscopy

To visualize cellular components, the fluorescent antibody-stained slides were imaged using an inverted Zeiss LSM 710 NLO Laser Scanning Confocal Microscope (Carl Zeiss, Jena, Germany). Nuclear targets were observed using a Plan-Apochromat 63x/(NA 1.4) DIC M27 oil immersion objective lens. The image scan area was 135.0 μm X 135.0 μm and 1,912 X 1,912 pixels (pixel size 0.07 μm) with a pixel dwell time of 0.68 μs and 12-bit sampling depth. The pinhole was set for optimal sectioning and step (~0.7 μm) with a refractive index correction of 1.46 to adjust for the mounting medium. We collected images of regions of interest (ROI) within 300 μm from the edge of each of three sections (n = 3) per experimental group.

The Z-stack and tilescan modules on the ZEN software (version 3.4, ZEISS) for confocal microscopy were used to acquire a 256.42 μm (2 x 2) region with optimal interval (0.32 μm) for a 7.06 μm stack with 23 slices. Optimizing for both speed and resolution, the “Smartest (Line)” setup was used for scanning of Alexa Fluor 488 and Alexa Fluor 633 on track 1; DAPI and Alexa Fluor 55 on track 2. DAPI was excited with a 405 nm Violet HeNe laser and emitted light was collected at 371–464 nm. Alexa Fluor 488 was excited with a 488 nm Argon multiline laser and emitted light was collected at 493–523 nm. Alexa Fluor 555 was excited with a 561 nm Green DPSS laser and emitted light was collected at 552–572 nm. Alexa Fluor 647 was excited with a 633 nm Red HeNe laser and emitted light was collected at 653–669 nm. The stacked confocal images were reconstructed and preprocessed using IMARIS (version 9.9, Bitplane). All measurements were made by a single observer who was blinded to the experimental groups to control for experimenter bias. Automated detection and segmentation of cell nuclei was done by opening the ImageJ Tool Labkit within Imaris. For nuclear foci counts, the sample size is shown as the number of randomly selected nuclei counted for each experimental group. We used unbiased stereology with disectors to count the cell-specific markers NEUN and MBP relative to DAPI-stained nuclei within each ROI. At least 200 cells were counted in all treatment groups. We also quantified the density of GFAP-stained processes with cycloids (to account for linear organization along the edge of the organoid) using STEPanizer [107].

### RNA-sequencing and analysis

XB C2 organoids were collected at 48 hours post irradiation for RNA-sequencing analysis. For each experimental group, three wells (containing 3–4 organoids per well) were collected for RNA extraction using the AllPrep DNA/RNA/miRNA Universal Kit (Qiagen #80224) according to the manufacturer’s instructions. Prior to library preparation, RNA quality was assessed using TapeStation 2200 and RNA ScreenTape (Agilent). RNA integrity number (RIN) values ranged from 9.0–9.8. RNA was quantitated using Qubit 4 fluorometer (Invitrogen). RNA libraries were prepared from 100 ng of total RNA using the Ovation^®^ Universal RNA-Seq System (NuGEN #0343) according to the manufacturer’s protocol. The RNA libraries were pooled and sequenced as 100 bpx2 paired-end reads, using the HiSeq 4000 system to a depth of 45 million reads per sample. The bcl2fastq (V.2.19.0.316) conversion software was used for demultiplexing and conversion of the Illumina sequencer output binary basecall (BCL) files to FASTQ files. The quality of raw sequencing reads was analyzed using FastQC. Trimmomatic was used to trim the reads and then the quality was assessed once again with FastQC. The reads were aligned to the human reference genome hg38 using STAR (version 2.6.1a). BAM files were also generated using STAR and analyzed using HTSeq counts and the Integrated Genome Viewer. The BAM files and gene counts were uploaded to usegalaxy.org for Reference-based RNA-seq data analysis [108]. Differential expression analysis was performed on the gene counts using DESeq2 (V2.11.40.7+galaxy1). PCA plot, heat maps, volcano plots, Gene Set Enrichment Analysis, Gene Ontology (GO) analysis, and KEGG pathway analysis were also generated using Galaxy. Ingenuity Pathway Analysis (IPA) version 81348237 was used to analyze differentially expressed genes, identify potential biomarkers, and recommend therapeutic targets.

### Statistics and reproducibility

The subject identifiers were blinded for the observer collecting images with confocal microscopy and performing quantitative analysis. Samples were divided into groups for control or irradiated (0.5 Gy, 2 Gy). The IF staining experiments were done using independently generated organoids from two different cell lines. The RNA sequencing of XB organoids included three biological replicates containing 3–4 organoids each for the control and irradiated groups. Statistical significance was established at adjusted p-value (FDR) < 0.05.

## Supporting information

S1 Table Significantly differentially expressed mitochondrial genes with FDR values, and log2FC values are shown. (DOCX)

S2 Table Additional information on the cell lines used in this study. (DOCX)

S1 Data (XLSX)

S1 Fig Expression of stem/progenitor and mature brain cell types. (A) Immunostaining and characterization of Day 38 WT83 C6 organoids showing (from left to right) gene expression of SOX2 and NEUN (first), MAP2 (second), OLIG2 (third), and NESTIN (last). (B) Immunostaining and characterization of WT83 C6 organoids showing (from left to right) gene expression of SOX2 and NEUN at Day 52 (first), MAP2 and OLIG2 at Day 55 (second), MBP and OLIG2 at Day 100 (third), and MAP2 and GFAP (last). (C) Representative image use for cell-type specific foci quantification (data are not provided since analysis is ongoing) of β-III-tubulin (immature and mature neurons) and NFH (mature neurons). The scale bar is 100 μm in panels A and B; 10 μm in panel C.
(TIF)

S2 Fig Representative trimmed FastQC. Read quality scores for RNA extracted from a batch of organoids in the control group.
(TIF)
