# Supplementary material for: Effects of acute low-moderate dose ionizing radiation to human brain organoids
Source: PLoS One. 2023 May 31;18(5):e0282958. doi: 10.1371/journal.pone.0282958 (PMC10231836; doi:10.1371/journal.pone.0282958)
Supplement: S1 Table — (DOCX) [file pone.0282958.s001.docx]

| Gene name | Function | FDR | log2(FC) |
| --- | --- | --- | --- |
| MT-CYB | cytochrome b | log2(FC) = -0.29 | FDR = 0.040 |
| MT-ATP6 | ATP synthase F0 subunit 6 | log2(FC) = -0.30 | FDR = 0.046 |
| MT-ATP8 | ATP synthase F0 subunit 8 | log2(FC) = -0.41 | FDR = 0.013 |
| MT-ND3 | NADH dehydrogenase subunit 3 | log2(FC) = -0.48 | FDR = 0.0021 |
| MT-ND5 | NADH dehydrogenase subunit 5 | log2(FC) = -0.32 | FDR = 0.031 |
| MT-CO1 | cytochrome c oxidase subunit I | log2(FC) = -0.33 | FDR = 0.038 |
| MT-CO3 | cytochrome c oxidase subunit III | log2(FC) = -0.37 | FDR = 0.027 |
| MT-ND4L | NADH dehydrogenase subunit 4L | log2FC = -0.40 | FDR = 0.021 |
| CEROX1 | cytoplasmic endogenous regulator of oxidative phosphorylation 1 | log2(FC) = -0.44 | FDR = 0.042 |

**S1 Table. Significantly differentially expressed mitochondrial genes with FDR values, and log2FC values are shown.**
