# Supplementary material for: Effects of acute low-moderate dose ionizing radiation to human brain organoids
Source: PLoS One. 2023 May 31;18(5):e0282958. doi: 10.1371/journal.pone.0282958 (PMC10231836; doi:10.1371/journal.pone.0282958)
Supplement: S2 Table — (DOCX) [file pone.0282958.s002.docx]

| **Line ID** | **Reprogramming** | **Cell source** | **Clinical Feature** | **Reference** |
| --- | --- | --- | --- | --- |
| WT83 C6 | Lentiviral | Fibroblast | Healthy | [KCC2 rescues functional deficits in human neurons derived from patients with Rett syndrome – PubMed (nih.gov)](https://pubmed.ncbi.nlm.nih.gov/26733678/) |
| XB C2 | Episomal | Peripheral blood | Healthy | [High-Level Precise Knockin of iPSCs by Simultaneous Reprogramming and Genome Editing of Human Peripheral Blood Mononuclear Cells – PubMed (nih.gov)](https://pubmed.ncbi.nlm.nih.gov/29754960/) |

**S2 Table. Additional information on the cell lines used in this study.**
