# Supplementary figures and images for: Effects of acute low-moderate dose ionizing radiation to human brain organoids
Source: PLoS One. 2023 May 31;18(5):e0282958. doi: 10.1371/journal.pone.0282958 (PMC10231836; doi:10.1371/journal.pone.0282958)

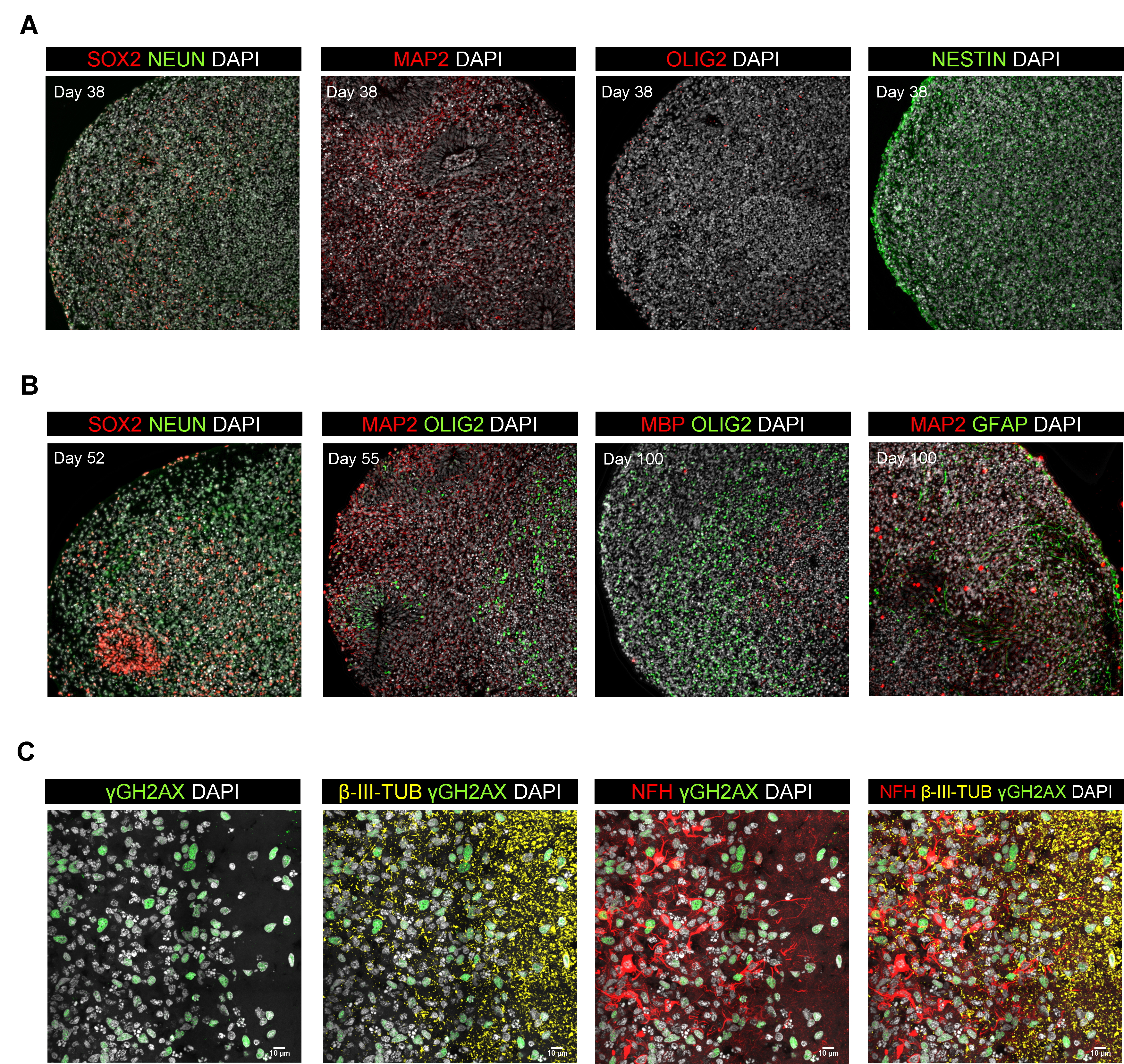

Supplement: S1 Fig — (A) Immunostaining and characterization of Day 38 WT83 C6 organoids showing (from left to right) gene expression of SOX2 and NEUN (first), MAP2 (second), OLIG2 (third), and NESTIN (last). (B) Immunostaining and characterization of WT83 C6 organoids showing (from left to right) gene expression of SOX2 and NEUN at Day 52 (first), MAP2 and OLIG2 at Day 55 (second), MBP and OLIG2 at Day 100 (third), and MAP2 and GFAP (last). (C) Representative image use for cell-type specific foci quantification (data are not provided since analysis is ongoing) of β-III-tubulin (immature and mature neurons) and NFH (mature neurons). The scale bar is 100 μm in panels A and B; 10 μm in panel C. (TIF) [file pone.0282958.s004.tif]

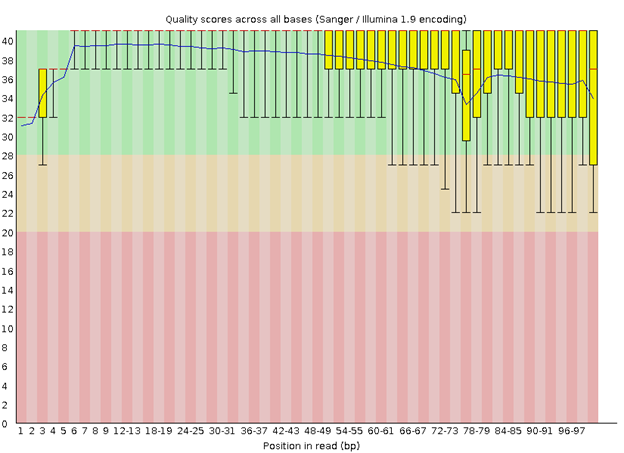

Supplement: S2 Fig — Read quality scores for RNA extracted from a batch of organoids in the control group. (TIF) [file pone.0282958.s005.tif]
